# Protective Effects of the Key Compounds Isolated from *Corni fructus* against β-Amyloid-Induced Neurotoxicity in PC12 Cells 

**DOI:** 10.3390/molecules170910831

**Published:** 2012-09-10

**Authors:** Seung-Young Hong, Woo-Sik Jeong, Mira Jun

**Affiliations:** 1 Department of Food Science and Nutrition, Dong-A University, Busan 604-714, Korea; Email: aseung02@hanmail.net; 2 Department of Food & Life Sciences, Inje University, Gimhae, Gyeongnam 621-749, Korea; Email: jeongws@inje.ac.kr

**Keywords:** Alzheimer’s disease (AD), amyloid β peptide (Aβ), *Corni fructus*, apoptosis, anti-oxidant, neuroprotection

## Abstract

β-Amyloid (Aβ) peptide is the major component of senile plaques and is considered to have a causal role in the development and progression of Alzheimer’s disease (AD). There is compelling evidence supporting the notion that Aβ-induced cytotoxicity is mediated though the generation of ROS. In the present study, we investigated the neuroprotective effects of ursolic acid (UA), *p*-coumaric acid (*p*-CA), and gallic acid (GA) isolated from *Corni fructus* (CF) against Aβ(25–35)-induced toxicity in PC12 cell. Exposure of PC12 cells to 50 μM Aβ(25–35) increased cellular oxidative stress, the number of apoptotic cells and caspase-3 activity and finally caused significant cell death. However, UA, *p*-CA, and GA not only suppressed the generation of ROS but also attenuated DNA fragmentation and eventually attenuated Aβ-induced apoptosis in a dose-dependent manner. In protecting cells against Aβ neurotoxicity, UA and GA possessed stronger ability against ROS generation than *p*-CA, while *p*-CA showed the strongest anti-apoptotic activity. Particularly, *p*-CA protected cells at the concentration range from 0.5 up to 125 μM without any adverse effect. Taken together, these effects of UA, *p*-CA, and GA may be partly associated with the neuroprotective effect of CF. Furthermore, our findings might raise a possibility of therapeutic applications of CF for preventing and/or treating neurodegenerative diseases.

## 1. Introduction

Alzheimer’s disease (AD) is a neurodegenerative disorder clinically characterized by progressive loss of memory and impairments in language and behavior [1]. The major neuropathological features in AD include neurofibrillary tangles and extracellular deposition of β-amyloid peptide (Aβ) [2]. The Aβ peptide is derived from amyloid precursor peptide (APP) though α-, β- and γ-secretase. The deposition of insoluble Aβ produces the aggregation of the peptide-forming amyloid fibrils that have been reported to be neurotoxic *in vitro* and *in vivo* [3]. Although the mechanisms of neuronal cell death in AD have not been fully elucidated, neurotoxicity exerted by Aβ may involve several mechanisms such as ROS generation, increase of intracellular Ca^2+^, and inflammation [4,5]. Many studies have confirmed that oxidative stress is involved in AD and the excessive production of Aβ itself leads to Aβ-induced free radical generation which leads to cell death [6].

Antioxidants have shown to be effective in preventing neurodegenerative disorders including Aβ-induced neurotoxicity [7,8]. Much attention has been paid on natural antioxidants with radical scavenging effect against oxidative damage such as resveratrol and tea catechins [9,10]. Therefore, searching for the compound attenuating oxidative stress might provide a therapeutic strategy to prevent and/or treat Aβ-induced neurotoxicity.

*Corni fructus* (CF) is the fruit of *Cornus officinalis* Sieb. et Zucc (Cornaceae). The fruit is considered one of the 25 plant-based drugs most frequently used in Eastern Asian countries such as China, Japan, and Korea. CF possesses antioxidative, antidiabetes, and antineoplastic effects [11,12,13]. It also has other effects such as anti-inflammation, hepatoprotection and *etc*. [14]. However, in spite of its diverse biological activities, the neuroprotective effects of CF have not been studied yet.

The EtOAc fraction of CF ethanol extract exhibited strong protective effect against Aβ(25–35)-induced injury in PC12 cells and three key compounds, ursolic acid (UA), *p*-coumaric acid (*p*-CA) and gallic acid (GA) were isolated in pure form by the activity-guided isolation in our previous study and identified [15]. Therefore, the aim of the present study was to investigate the neuroprotective properties of the key compounds isolated from CF against Aβ(25–35)-induced injury.

## 2. Results and Discussion

### 2.1. Ursolic Acid, p-Coumaric Acid and Gallic Acid Protected PC12 Cells against Aβ(25–35)-Induced Cytotoxicity

**Figure 1:**
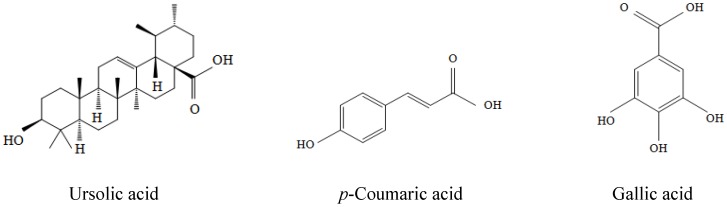
The chemical structures of the key compounds isolated from ethyl acetate fraction of *Corni fructus.*

**Figure 2:**
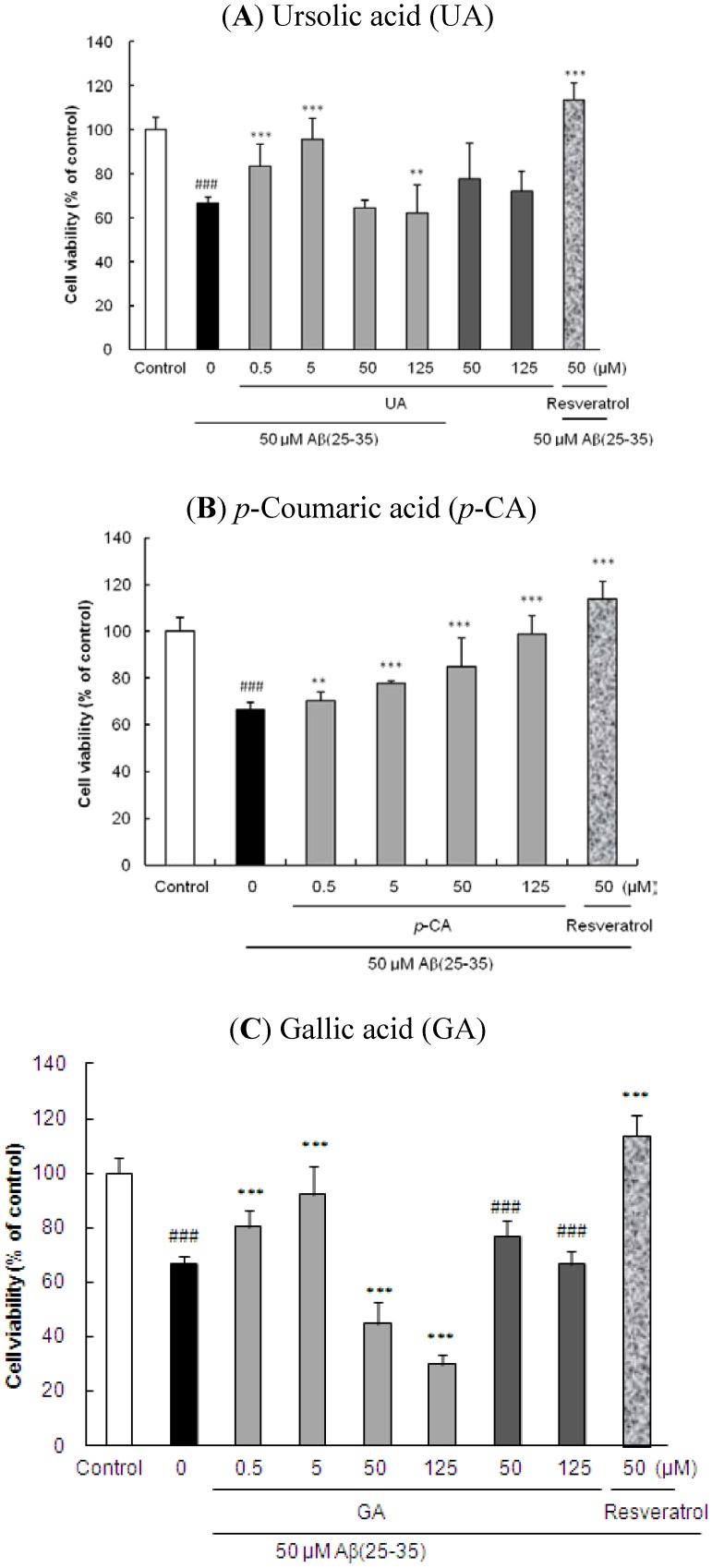
Protective effects of key compounds isolated from *Corni fructus* on Aβ(25–35)-induced cell death. PC12 cells were pretreated with (**A**) UA, (**B**) *p*-CA, and (**C**) GA for 1 h and further treated with 50 μM of Aβ(25–35) for 24 h. Cell viability was assessed by measuring MTT reduction. ^### ^*p <* 0.001 and ^## ^*p <* 0.01 *versus* control group. *** *p <* 0.001 and ** *p <* 0.01 *versus* the group treated with Aβ(25–35) alone.

The chemical structures of UA, *p*-CA, and GA are presented in Figure 1. To assess Aβ(25–35)-induced neuronal cell death, the MTT reduction assay was performed. The concentration of 50 μM was used for determining Aβ(25–35)-induced cellular damage based on our previous results [16]. As shown in Figure 2, PC12 cells treated for 24 h with 50 μM Aβ(25–35) had cell viability reduction of 66.55 ± 2.84% compared to the control (*p <* 0.001). UA showed the strongest inhibitory activity against Aβ(25–35)-induced cell death followed by GA and *p*-CA at 0.5 and 5 μM. However, treatment of UA at 50 and 125 μM showed somewhat adverse effects on neuronal cell viability. To investigate whether UA itself had toxicity or not, PC12 cells were exposed to 50 and 125 μM UA without 50 μM Aβ(25–35) treatment. The result showed that the viability of UA-treated PC12 cells at 50 and 125 μM decreased in comparison to the control group but UA exerted neither protection nor exacerbation against 50 μM Aβ(25–35)-induced neurotoxicity at the concentrations of 50 and 125 μM (Figure 2A). Pretreatment of the cells with *p*-CA also reduced the neuronal cell death caused by Aβ in concentration-dependent manner (Figure 2B). Particularly, *p*-CA at the concentration of 125 μM (98.89 ± 7.61%, *p <* 0.001) showed almost complete inhibition of the Aβ(25–35)-induced cell death. Moreover, the potency of 250 μM *p*-CA (112.50 ± 4.72%) was similar to that of 50 μM resveratrol (113.63 ± 7.57%) without significant difference (*p* < 0.001, data not shown). Here, we compared the key compounds from CF with resveratrol, a potent anti-dementia agent in terms of neuroprotective actions. Among these key compounds, only *p*-CA alone did not affect cell viability even at the higher concentration (>125 μM) (data not shown).

GA also was found to possess similar effect as UA on neuronal cell survival at the concentrations higher than 50 μM. High concentration of GA itself (>50 μM) exerted neuronal cell death showed 19.81 ± 0.84% of MTT reduction rate (Figure 2C). Similar result was found in the study of Ban *et al*. [17]. However, low concentrations (0.5 and 5 μM) of UA and GA did not affect cell viability (data not shown).

### 2.2. Ursolic Acid, p-Coumaric Acid and Gallic acid Protected PC12 Cells against Aβ(25–35)-Induced Intracellular ROS Accumulation in PC12 Cells

To determine whether the compounds from CF attenuate cell death by blocking ROS generation or not, the intracellular ROS concentration was measured using the CM-H_2_DCFDA fluorescence dye. In previous MTT reduction experiment, we have demonstrated that UA and GA had adverse effect on cell viability at the concentration over 50 μM. Therefore, for further experiments, the experimental concentration ranges of UA and GA were adjusted from 0.5 to 5 μM that didn’t affect cell viability. 

**Figure 3:**
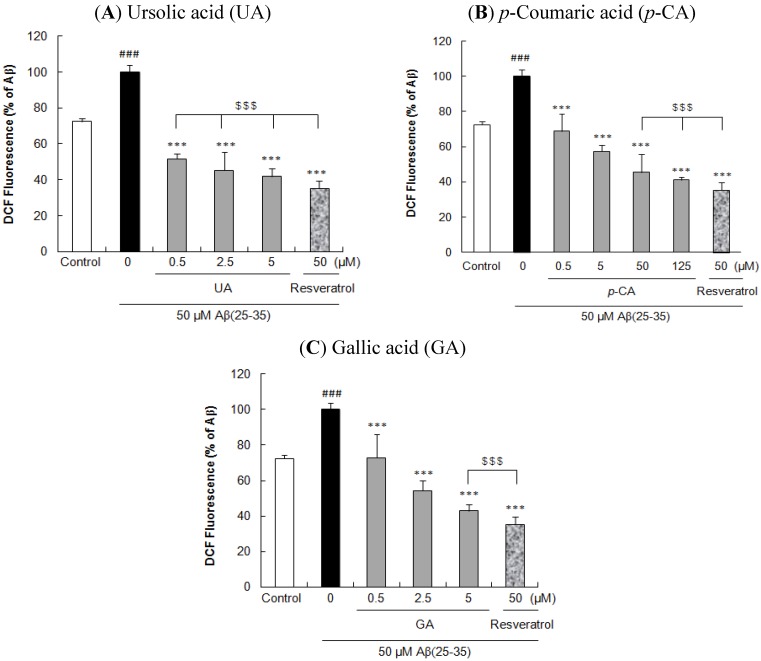
Inhibitory effects of key compounds isolated from *Corni fructus* on Aβ(25–35)-induced intracellular ROS accumulation. PC12 cells were pretreated with (**A**) UA, (**B**) *p*-CA, and (**C**) GA for 1 h and further treated with 50 μM of Aβ(25–35) for 24 h. ROS production was measured using the CM-H_2_DCFDA fluorescent dye. ^### ^*p <* 0.001 *versus* control group. *** *p <* 0.001 *versus* the group treated with Aβ(25–35) alone. $$$, not significantly different.

Exposure of PC12 cells treated with Aβ(25–35) resulted in a significant increase of ROS levels (72.41 ± 1.81% *vs.* 100 ± 3.83%, Aβ(25–35) *vs.* control, *p* < 0.001, Figure 3). However, treatment of cells with the isolated compounds before exposure to Aβ(25–35) appreciably inhibited the ROS generation in dose-dependent manner. At the concentration of 0.5 μM, UA, *p*-CA, and GA significantly reduced Aβ(25–35)-induced ROS generation (51.69 ± 2.88%, 68.86 ± 9.69%, and 73.00 ± 13.05%, respectively, *p* < 0.001). In particular, UA showed the strongest inhibitory activity on the Aβ(25–35)-induced ROS accumulation followed by GA and *p*-CA at the low concentrations (0.5 and 5 μM). Furthermore, UA possessed similar ROS protective effect with resveratrol without significant difference (*p* < 0.001). Moreover, the inhibitory effect of 50, 125 μM *p*-CA (45.35 ± 10.48% and 41.11 ± 1.58%, respectively), and 5 μM GA (42.95 ± 3.64%) were similar to that of 50 μM resveratrol (35.31 ± 4.30%) without significant difference. UA, *p*-CA, and GA did not show direct reaction with CM-H_2_DCFDA to generate fluorescence (data not shown).

Abnormal production of Aβ is a primary event in the pathological cascade of AD [18]. Several experimental studies suggest an association between Aβ, oxidative stress and apoptosis with AD [19,20]. ROS produced in mitochondria may leak to the cytoplasm, leading to oxidative stress and the initiation of apoptosis [21,22] Several antioxidants have been demonstrated to be useful for attenuation of AD development [23,24,25].

Oxidative damage occurs when ROS are overproduced and exceeds the capacity of the endogenous antioxidant defense systems [26]. Several lines of evidence support the involvement of oxidative stress as an active factor in Aβ-mediated neuropathology, by facilitating neurodegeneration though a wide range of molecular events that disturb neuronal homeostasis [27]. Here, we found that UA, *p*-CA, and GA strongly suppressed the accumulation of intracellular ROS in PC12 cells. The result have confirmed that Aβ(25–35)-induced PC12 cells produce ROS and can be protected from Aβ toxicity by antioxidant such as UA, *p*-CA, and GA. From our results, it can be explained that cytoprotective effects of UA, *p*-CA, and GA may be attributed, at least in part, to their antioxidant properties.

### 2.3. Ursolic Acid, p-Coumaric Acid and Gallic Acid Protected PC12 Cells against Aβ(25–35)-Induced Apoptosis

**Figure 4:**
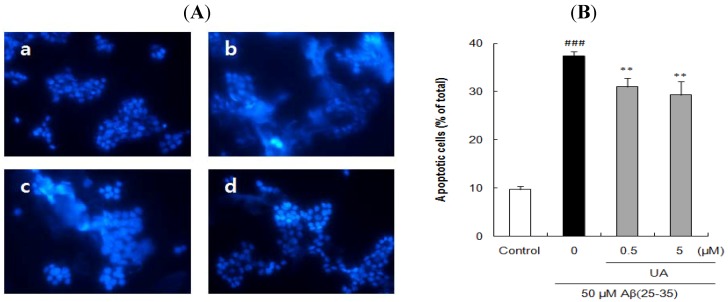
Inhibitory effects of ursolic acid isolated from *Corni fructus* on Aβ(25–35)-induced apoptosis. (**A**) PC12 cells were pretreated with UA for 1 h and further treated with 50 μM of Aβ(25–35) for 24 h. Morphological apoptosis was determined by Hoechst 33342 staining under fluorescence microscopy (magnification ×400). (**a**) Control; (**b**) 50 μM Aβ(25–35); (**c**) 50 μM Aβ(25–35) + 0.5 μM UA; (**d**) 50 μM Aβ(25–35) + 5 μM UA. (**B**) Histogram showing the percentage of apoptotic cells in total cell population after different treatments. ^### ^*p <* 0.001 *versus* normal group. ** *p <* 0.01 *versus* the group treated with Aβ(25–35) alone. Data represent the mean ± SD of three independent experiments.

**Figure 5:**
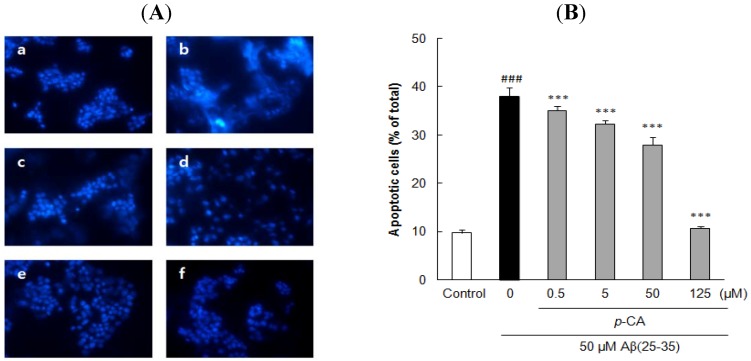
Inhibitory effects of *p*-coumaric acid isolated from *Corni fructus* on Aβ(25–35)-induced apoptosis. (**A**) PC12 cells were pretreated with *p*-CA for 1 h and further treated with 50 μM of Aβ(25–35) for 24 h. Morphological apoptosis was determined by Hoechst 33342 staining under fluorescence microscopy (magnification ×400). (**a**) Control; (**b**) 50 μM Aβ(25–35); (**c**) 50 μM Aβ(25–35) + 0.5 μM *p*-CA; (**d**) 50 μM Aβ(25–35) + 5 μM *p*-CA; (**e**) 50 μM Aβ(25–35) + 50 μM *p*-CA, (f) 50 μM Aβ(25–35) + 125 μM *p*-CA. (**B**) Histogram showing the percentage of apoptotic cells in total cell population after different treatments. ^### ^*p <* 0.001 *versus* normal group. *** *p <* 0.001 *versus* the group treated with Aβ(25–35) alone. Data represent the mean ± SD of three independent experiments.

**Figure 6:**
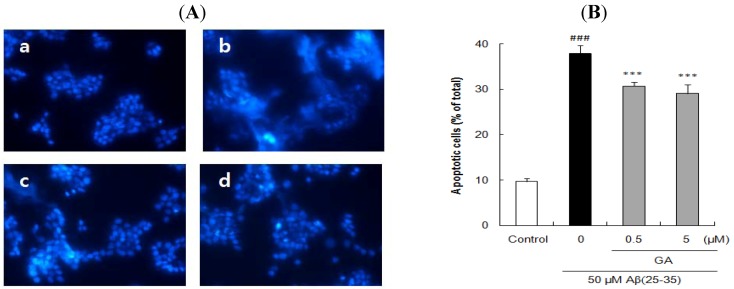
Inhibitory effects of gallic acid isolated from *Corni fructus* on Aβ(25–35)-induced apoptosis. (**A**) PC12 cells were pretreated with GA for 1 h and further treated with 50 μM of Aβ(25–35) for 24 h. Morphological apoptosis was determined by Hoechst 33342 staining under fluorescence microscopy (magnification ×400). (**a**) Control; (**b**) 50 μM Aβ(25–35); (**c**) 50 μM Aβ(25–35) + 0.5 μM GA; (d) 50 μM Aβ(25–35) + 5 μM GA. (**B**) Histogram showing the percentage of apoptotic cells in total cell population after different treatments. ^### ^*p <* 0.001 *versus* normal group. *** *p <* 0.001 *versus* the group treated with Aβ(25–35) alone. Data represent the mean ± SD of three independent experiments.

The cells stained with Hoechst 33342 revealed marked chomatin condensation and apoptotic body formation when examined by a fluorescence microscope. In contrast to round and blue nuclei observed in viable PC12 cells of control group (Figure 4Aa, Figure 5Aa, and Figure 6Aa), several marked characteristics of apoptosis such as chromatin condensation and nuclear fragmentation were observed in cells exposed to 50 μM Aβ(25–35) for 24 h (Figure 4Ab, Figure 5Ab, and Figure 6Ab). These characteristic changes in cellular morphology were significantly reduced in the cells pretreated with the compounds (Figure 4, Figure 5 and Figure 6). Especially, the preincubation with 125 μM *p*-CA almost completely blocked the apoptosis in terms of the morphological appearance of PC12 cells (Figure 5Af).

The proportion of apoptotic neurons was calculated in Figure 4B, Figure 5B, and Figure 6B. In comparison to only 9.72 ± 0.70% apoptotic cells in total population of PC12 cells in control group, exposure to 50 μM Aβ(25–35) alone produced 37.81 ± 1.82% apoptosis. However, pre-incubation with 0.5 and 5 μM of UA or GA significantly decreased the Aβ(25–35)-induced apoptosis. Moreover, the addition of *p*-CA significantly decreased Aβ(25–35)-induced apoptotic cell death showing 35.10 ± 0.81%, 32.27 ± 0.76%, and 27.94 ± 1.61% at the concentration of 0.5, 5, and 50 μM. In particular, *p*-CA at the concentration of 125 μM (10.65 ± 0.36%, *p <* 0.001) showed almost complete inhibition of the Aβ(25–35)-induced apoptosis. The neuroprotective effects of isolated compounds from CF against cellular apoptosis displayed dose-dependent pattern.

Several investigators have demonstrated that the neurotoxicity of Aβ can be mediated by ROS, which may contribute to the increased evidence of apoptosis found in AD [28,29]. Aβ-induced ROS accumulation cause damage to neuronal membrane lipids, proteins, and nucleic acids, and ultimately leads to apoptosis which is believed to play a critical role in cell loss during progression of AD [30,31]. Some of the classical features of Aβ-induced apoptosis such as decreased cell viability, DNA condensation, and DNA fragmentation were detected in PC12 cells in this study.

### 2.4. Ursolic Acid, p-Coumaric Acid and Gallic Acid Inhibited Aβ(25–35)-Induced Caspase-3 Activity

**Figure 7:**
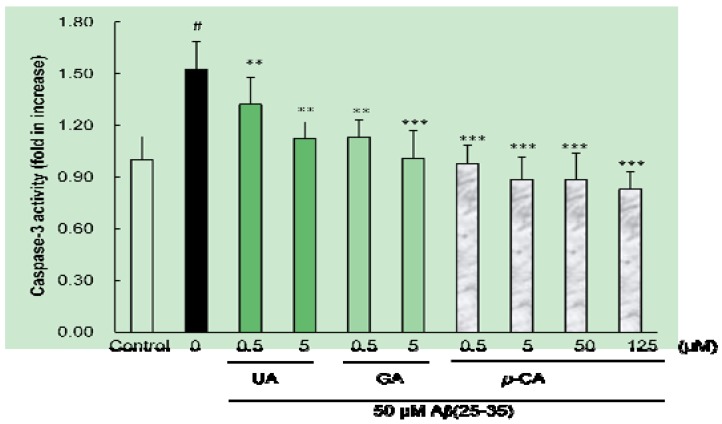
Protective effects of *p*-coumaric acid isolated from *Corni fructus* on Aβ(25–35)-induced caspase-3 activation. PC12 cells were pretreated with *p*-CA for 1h and further treated with 50 μM of Aβ(25–35) for 24 h. Caspase-3 activity was measured with the colorimetric caspase-3 assay kit. ^# ^*p <* 0.05 *versus* normal group. *** *p <* 0.001 *versus* the group treated with Aβ(25–35) alone.

The activation of caspase-3, a family of cysteine proteases, is believed to be an important factor for apoptosis [31]. To examine whether the isolated compounds modulates the proteolytic activity of caspase-3, we measured the caspase-3-like protease activity using the substrate DEVD-*p*NA. As shown in Figure 7, the enzyme activity of caspase-3 was significantly elevated in Aβ(25–35)-treated PC12 cells (1.52 fold, *p* < 0.05).

However, preincubation with UA and GA for 1 h attenuated Aβ(25–35)-induced caspase-3 activation. Among the three isolated compounds, *p*-CA exhibited the strongest inhibitory effect on caspase-3 activity even at the concentration of 0.5 μM.

Apoptosis is associated with the activation of a family of aspartic acid-specific cysteine proteases [32,33]. The present study demonstrated that Aβ induces the apoptotic death of PC12 cells, as judged by morphological nuclear changes, DNA fragmentation, and cell blebbing. These apoptotic features were attenuated by addition of UA, *p*-CA, and GA, suggest that these compounds exert protective effects against Aβ-induced cell death by affecting partly by attenuating caspase-3 activity. In protecting cells against Aβ-induced apoptosis, UA and GA showed similar potency, while *p*-CA was stronger than UA and GA. Furthermore, in the present study, it was shown that *p*-CA can inhibit caspase-3 activity in Aβ (25–35)-treated PC12 cells, suggesting that *p*-CA could exert a protective role at the execution phase of apoptosis. Furthermore, these results suggest a special link between ROS and apoptosis, and the protective effect of UA, *p*-CA and GA on apoptosis is due, at least in part, to its antioxidant property.

The key compounds isolated from CF have the specific characteristics. UA, a natural pentacyclic triterpenoid carboxylic acid, is the major component of some traditional medicinal herbs and is well known to possess a wide range of biological functions, such as antioxidative, anti-inflammation and anticancer activities, that are able to counteract endogenous and exogenous biological stimuli [34,35]. A study on mechanism revealed that UA blocked the cell cycle progression in the G1 phase and that UA treatment resulted in the triggering of apoptosis as determined by a DNA fragmentation assay [36]. 

*p*-CA found in fruits and vegetables is an intermediate product of the phenylpropanoid pathway in plants. Previous investigators have shown that *p*-coumaric acid exhibited antioxidant and anti-inflammatory properties [37,38]. Moreover, maltolyl *p*-coumarate, a *p*-CA derivative, is a potentially effective candidate against AD [39]. The ethylenic group between a phenyl ring carrying a *p*-hydroxyl group and the carboxylate group has a highly favorable effect on the reducing properties of the OH group [40,41]. Moreover, the presence of the –CH=CH–COOH groups in *p*-CA ensures greater subsequent radical stabilization [40]. Therefore, *p*-CA is very important for antioxidant activity, which can be boosted by the electron-donating substituent, the –CH=CH–COOH, which leads *p*-CA to be beneficial inhibitors of ROS accumulation and cell protection. 

GA, a strong natural antioxidant, is widespread in tea and wine and has been proven to be one of the anticarcinogenic polyphenols present in green tea [42]. Due to this antioxidant effect, GA containing plant extracts have showed antidiabetic, antiangiogenic, and antimelanogenic effects and reduced oxidative liver and kidney damage [17]. From its unique structure, GA is possible to obtain two radicals by abstraction of a hydrogen atom from the 3-OH (5-OH) and 4-OH groups [43]. Overall, the resulting effect of key compounds with the characteristics such as the structure-activity relationships and other abilities may help to elucidate this issue or even to discover new neuroprotective agents in further studies.

Exposure to Aβ leads to the accumulation of ROS, leading to subsequent caspase-3 activation, cell apoptosis and neuronal cell death. In the present study, we have shown that UA, *p*-CA, and GA isolated from CF not only decreased Aβ(25–35)-induced ROS overproduction and caspase-3 activity, but also attenuated apoptosis and cell death in PC12 cells. The results suggest that these compounds are novel and effective neuroprotective agents against oxidative damage in Aβ(25–35)-induced toxicity. In protecting cells against Aβ neurotoxicity, UA and GA have stronger ability against ROS generation than *p*-CA, while *p*-CA showed the strongest anti-apoptotic activity among the key compounds isolated from CF. Particularly, *p*-CA also protects PC12 cells up to 125 μM concentration, the higher concentration that we utilized without any cytotoxicity. Further, exploration of UA, *p*-CA, and GA antioxidant properties may provide opportunities for novel pharmacological interventions aiming at preventing and/or palliating the consequences of AD. 

## 3. Experimental

### 3.1. General

β-Amyloid [Aβ(25–35)], 3,5,4'-trihydroxy-*trans*-stilbene (resveratrol), and 3-[4,5-dimethylthiazol-2-yl]-2,5-diphenyltetrazolium bromide (MTT) were purchased from Sigma Chemical Co. (St. Louis, MO, USA). RPMI 1640 medium, phosphate buffered saline (PBS), trypsin 0.25% solution, and penicillin/streptomycin solution were obtained from Hyclone Laboratories, Inc. (Logan, UT, USA). Fetal bovine serum was obtained by PAA Laboratories GmbH. (Linz, Austria). N2 supplement and RPMI 1640 phenol red free medium were purchased from Gibco BRL (Grand Island, NY, USA). 5-[and-6]-Chloromethyl-2',7'-dichlorodihydrofluorescein diacetate, acetyl ester (CM-H_2_DCFDA) and Hoechst 33342 dye were obtained from Molecular Probes Inc. (Eugene, OR, USA). Dimethylsulfoxide (DMSO) was supplied from Bio Basic Inc. (Ontario, Canada). All other chemicals used were of the highest grade commercially available. Aβ(25–35), which is the most toxic peptide fragment derived from APP, was dissolved in DMSO at a concentration of 10 mM and stored at −20 °C. The stock solution was diluted with PBS to appropriate concentration and incubated for 48 h at 37 °C for aggregation prior to each experiment [16]. Samples were dissolved in DMSO and further diluted with sterilized water. The final concentration of DMSO was less than 0.01%, which did not affect cell viability.

### 3.2. Plant Material, Extraction, Fractionation, and Isolation of Key Compounds from Corni fructus

CF was purchased from agricultural farm in Gurye, Jeonnam, Korea in Nov. 2007. The voucher specimen (No. NFF 0407-002) has been deposited at Nutraceuticals and Functional Food Lab., Dong-A University, Busan, Korea and the whole plant extract was prepared as published earlier [15]. In brief, the dried CF (3 kg) was refluxed with 95% ethanol (11 L × 3) and its extract was evaporated to dryness. The ethanol extract (1.15 kg) was suspended in water, and partitioned successively with *n*-hexane, dichloromethane (MC), ethyl acetate (EtOAc), and *n*-butanol (BuOH) to give fractions of *n*-hexane (23 g), MC (89 g), EtOAc (110 g), and BuOH (253 g), respectively, and a water layer (680 g).

The EtOAc soluble fraction, which exhibited the most potent neuroprotective effect against Aβ (25–35)-induced neurotoxicity among the other organic solvent fractions, was applied onto silica gel (70–230 mesh Merck, Darmstadt, Germany) open column chomatography and five fractions including compound CO-**1** (20.3 mg) were obtained. The experiment of the activity-guided separation of fractions on neuroprotective effect against Aβ(25–35)-induced cell death showed that fraction 2 was more potent than others (data not shown), and so fraction 2 (565.5 mg) was separated by re-chomatography on a silica gel column with a *n*-hexane:EtOAc:MeOH step-gradient (20:10:1→10:10:1→5:10:1→0:0:1) to yield compound CO-**1** (3.1 mg), CO-**2** (10.7 mg) and compound CO-**3** (16.6 mg) and the chemical structure of the isolated compounds were identified as ursolic acid, *p*-coumaric acid, and gallic acid (Figure 1), respectively, in our previous study [15]. 

### 3.3. Cell Culture

Rat PC12 cell line, which has been used for neurological experiment, was obtained from the American Type Cell Collection (ATCC) and were maintained in RPMI 1640 medium supplemented with 10% donor equine serum, 5% fetal bovine serum, and 100 U/mL penicillin and 100 μg/mL streptomycin at 37 °C in a humidified 95% air/5% CO_2_ incubator. All cells were cultured in collagen-coated culture dishes. The medium was changed every other day, and cells were plated at an appropriate density according to each experimental scale. After 24 h subculture, cells were switched to serum-free N2 defined medium for treatment. 

### 3.4. Cell Viability Assay

Cell viability was assessed by measuring formazan produced by the reduction of MTT. The MTT assay is a sensitive measurement of the normal metabolic status of cells, particularly those of mitochondria, which reflects early cellular redox changes. Therefore, the amount of formazan produced is proportional to the number of viable cells. Briefly, the cells were plated in 96 well culture plates at the density of 1 × 10^4^ cells/well and allowed to adhere at 37 °C for 24 h. After incubation with various concentration of sample solution for 1 h, the cells were treated with 50 μM Aβ(25–35) for 24 h. Thereafter, MTT reagent (final concentration, 0.5 mg/mL) was added to each of the wells and the plate was incubated for an additional about 3 h at 37 °C. At the end of the incubated period, the medium with MTT was removed and 100 μL DMSO was added to each well. The formazan reduction product was measured by reading absorbance at 570 nm in a microplate reader (ELX808, Biotek, Winooski, VT, USA). Cell viability was expressed as a percentage of the control culture. 

### 3.5. Intracellular Reactive Oxygen Species Production Determination

Intracellular ROS was monitored by using the fluorescent probe 5-[and-6]-chloromethyl-2',7'-dichlorodihydrofluorescein diacetate (CM-H_2_DCFDA), which is intracellular oxidized to the fluorescent 2',7'-dichlorofluorescein (DCF) in the presence of cellular peroxides [44]. Briefly, PC12 cells were seeded at a density of 1 × 10^4^ cells/well into 96 well plates and allowed to grow at 37 °C for 24 h. After treatment with 50 μM Aβ(25–35) for 24 h in the presence or absence of sample solution, the serum-free N2 defined medium was switched to HBSS with 10 μM CM-H_2_DCFDA at 37 °C for 30 min. Then, the medium was removed and 100 μL HBSS was added to each well. Fluorescence intensity was measured at an excitation wavelength of 485 nm and an emission wavelength of 528 nm using a fluorescence spectrophotometer (FLX800, Biotek). Data were given as percents relative to the oxidative stress of the group treated with Aβ(25–35) alone set to 100%. 

### 3.6. Apoptotic Morphology Observation by Hoechst 33342 Staining

Chomosomal condensation and morphological changes in the nucleus were observed by using the chomatin dye, Hoechst 33342 [23]. Cells with homogeneously stained nuclei were considered to be viable. On the other hand, chomatin condensation and/or fragmentation were the characteristic indicative of apoptosis. Briefly, PC12 cells (1 × 10^6^) cultured on cover slips were plated in 6 well plates with 2 mL of medium in every well, and exposed to 50 μM Aβ(25–35) with various concentrations of sample solution for 24 h. After treatments, the cells were fixed with 4% formaldehyde in PBS for 20 min at RT and stained with 1 μg/mL Hoechst 33342 for 20 min at RT and Hoechst-stained cells were visualized and photographed under a fluorescent microscope (Olympus, Tokyo, Japan). To quantify the apoptotic process, neurons with fragmented or condensed DNA and normal DNA were counted. Data was shown as apoptotic cells as a percentage of total cells.

### 3.7. Measurement of Caspase-3 Activity

The extent of caspase-3 activation in PC12 cells treated with Aβ(25–35) was assessed using the commercially available colorimetric assay kit in accordance with the protocol supplied by the manufacturer (BioVision, Mountain View, CA, USA). The assay is based on spectrophotometric detection of the chomophore *p*-nitroanilide (*p*NA) after its cleavage by caspase-3 from the labeled substrate, DEVD-*p*NA. Briefly, cultured PC12 cells (1.5 × 10^6^) were lysed for 10 min in an ice bath, and the lysates were centrifuged at 10,000 g at 4 °C for 5 min. The protein concentrations in the supernatants were determined using the Bradford protein assay kit (Pierce Biotechnology, Rockford, IL, USA). Protein samples were incubated with the substrate peptide (200 μM) in 50 μL of incubation buffer at 37 °C for 1 h. Absorbance of the chomophore *p*NA produced was measured using a microplate reader at wavelength of 405 nm. Data were expressed as a fold increase in caspase activity of apoptotic cells over that of non induced cells. 

### 3.8. Statistical Analysis

All data were expressed as means ± standard deviation (SD). Statistical differences between groups were performed by analysis of variance (ANOVA) with Duncan’s multiple range test using the Statistical Analysis System (SAS). A value of *p <* 0.05 was considered statistically significant.

## 4. Conclusions

The results indicate, for the first time, that UA, *p*-CA and GA derived from CF exert a combination of neuroprotective, anti-oxidative and anti-apoptotic effects against Aβ(25–35) damage. On these grounds, UA, *p*-CA, and GA derived from CF demonstrated to have neuroprotective effect against Aβ(25–35)-induced neurotoxicity via anti-oxidant and anti-apoptotic property, could be useful as a therapeutic agent for treatment of Aβ-induced neuronal degeneration diseases such as AD. 
